# Strong whistler mode waves observed in the vicinity of Jupiter’s moons

**DOI:** 10.1038/s41467-018-05431-x

**Published:** 2018-08-07

**Authors:** Y. Y. Shprits, J. D. Menietti, A. Y. Drozdov, R. B. Horne, E. E. Woodfield, J. B. Groene, M. de Soria-Santacruz, T. F. Averkamp, H. Garrett, C. Paranicas, D. A. Gurnett

**Affiliations:** 1 Helmholtz Centre Potsdam, GFZ German Research Centre for Geosciences, Potsdam, 14473 Germany; 2 Institute for Physics and Astronomy, Universität Potsdam, Potsdam, 14469 Germany; 3 Department of Earth, Planetary, and Space Sciences, University of California, Los Angeles, 90095 CA USA; 4 Department of Physics and Astronomy, University of Iowa, Iowa City, 52242 IA USA; 5 British Antarctic Survey, Cambridge, CB3 0ET UK; 6 Jet Propulsion Laboratory, California Institute of Technology, Pasadena, 91109 CA USA; 7 Applied Physics Laboratory, Johns Hopkins University, Laurel, 20723 MD USA

## Abstract

Understanding of wave environments is critical for the understanding of how particles are accelerated and lost in space. This study shows that in the vicinity of Europa and Ganymede, that respectively have induced and internal magnetic fields, chorus wave power is significantly increased. The observed enhancements are persistent and exceed median values of wave activity by up to 6 orders of magnitude for Ganymede. Produced waves may have a pronounced effect on the acceleration and loss of particles in the Jovian magnetosphere and other astrophysical objects. The generated waves are capable of significantly modifying the energetic particle environment, accelerating particles to very high energies, or producing depletions in phase space density. Observations of Jupiter’s magnetosphere provide a unique opportunity to observe how objects with an internal magnetic field can interact with particles trapped in magnetic fields of larger scale objects.

## Abstract (web summary)

Observations of Jupiter’s magnetosphere provide opportunities to understand how magnetic fields interact with particles. Here, the authors report that the chorus wave power is increased in the vicinity of Europa and Ganymede. The generated waves are able to accelerate particles to very high energy.

## Introduction

Jupiter is 5.2 Astronomical Units (AU) from the Sun and has a magnetic moment 20,000 times higher than Earth. While the dynamics of the magnetosphere of Earth are driven by the solar wind, the Jovian magnetosphere is believed to be internally driven by the interchange instability which results from the loading of hot plasma from volcanic eruptions on Io. Jupiter’s magnetosphere is a unique natural laboratory that allows us to study its interactions with gravitationally bound moons and their internal or induced magnetic fields. Observations of Jupiter’s wave and particle environments near the moons allows us to directly observe how smaller magnetized or magnetically active objects interact with larger scale magnetospheric systems, and to understand the physics behind these interactions.

Galileo spacecraft measurements showed that Ganymede, located at ~15 *R*_J_ (*R*_J_ –Jupiter radii), has an internally produced magnetic field with a surface equatorial field strength of ~720 nT^1^. Observations of the wave environment further showed that plasma and radio waves are enhanced in the vicinity of Ganymede. Measurements during the first Ganymede flyby on 27 June 1996 revealed strong whistler mode waves^2^. These intense waves were only observed within ~4 radii of Ganymede, indicating that the generation of strong waves was related to the proximity to Ganymede.

In the Earth’s magnetosphere, which is well sampled and better understood, it has been established that chorus whistler mode plasma waves play a crucial role in the acceleration of electrons to relativistic energies, e.g., refs. ^3–6^. Similar mechanisms have been discussed and studied for Jupiter^7,8^. Depending on the distribution of waves in space, frequency and wave-normal angles, waves can either accelerate particles to relativistic and ultra-relativistic energies or produce a fast loss of particles. Strong waves associated with moons have been observed in the Kronian magnetosphere. Moons can cause loss of particles due to sweeping and absorption effects^9^, but can also produce a strong loss by generating waves that scatter particles in pitch angle^10^.

Our current study shows that induced waves ~1 nT in wave amplitude are strong enough to produce significant modifications to the wave and particle environment. Induced waves may either scatter particles and produce strong pitch angle diffusion^11,12^, or cause acceleration to relativistic or ultra-relativistic energies.

## Results

### Statistical comparison of flyby amplitudes and encounters

**Fig. 1:**
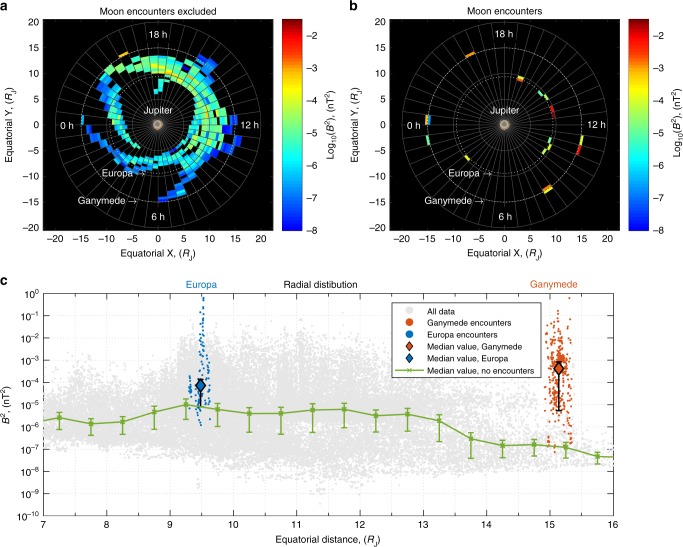
Galileo mission wave power statistics: **a** Average chorus intensity in each bin (Δ*R*_J_ = 0.5, ΔMLT = 0.5 h) for the equatorial region from 27 June 1996 until 5 November 2002, excluding encounters with the moons. **b** Same as **a** but for encounters with Europa (~9.4 *R*_J_) and Ganymede (~15 *R*_J_), positive x is toward the Sun (noon MLT), axes are in the magnetic equatorial plane. **c** Scatter plot of wave power as a function of the distance from Jupiter. Blue dots show Galileo encounters with Europa. Red dots show encounters with Ganymede. Diamond points are median values during encounters. Green line shows median values of the wave power excluding encounters. The error bars are absolute mean deviation. Median wave power during the encounters for Ganymede and Europa significantly exceeds the median values at corresponding distances from Jupiter

While only chorus wave observations from the first encounter with Ganymede have been reported and discussed in the scientific literature^2^, there were a number of passes where Galileo intercepted the orbits of Europa and Ganymede in close flybys (Fig. 1a). Galileo’s orbit provides sufficient orbital coverage to infer the radial profile of wave intensity (Fig. 1b) and to compare these intensities with averages during both Europa and Ganymede flybys. While there is a significant spread in the observed values of typical waves observed in Jupiter’s magnetosphere, the average amplitudes of intensities are on the scale of 10 pT^13^. There is also very little radial dependence of wave amplitudes, with a gradual decrease beyond 13 Jupiter radii. Measurements of wave power during Ganymede encounters show that the maximum wave power during encounters exceeds the most commonly observed wave power (median wave power) around 15 *R*_J_ by up to 6 orders of magnitude. If we assume that even 1% of waves propagate into the region of trapped population, such an increase in wave power will produce 10^4^ times faster loss or acceleration in the vicinity of the moons than in the surrounding environment, which is likely to produce significant loss or acceleration near the gas giant.

The increase in wave power for Europa is less pronounced, but most of the values lie well above the statistical averages (Fig. 1c). Note that maximum values of wave amplitude may have been missed due to a limited number of flybys, and maximum wave power very close to Ganymede that was not sampled may be even higher.

### Free energy for the excitation of waves

**Fig. 2:**
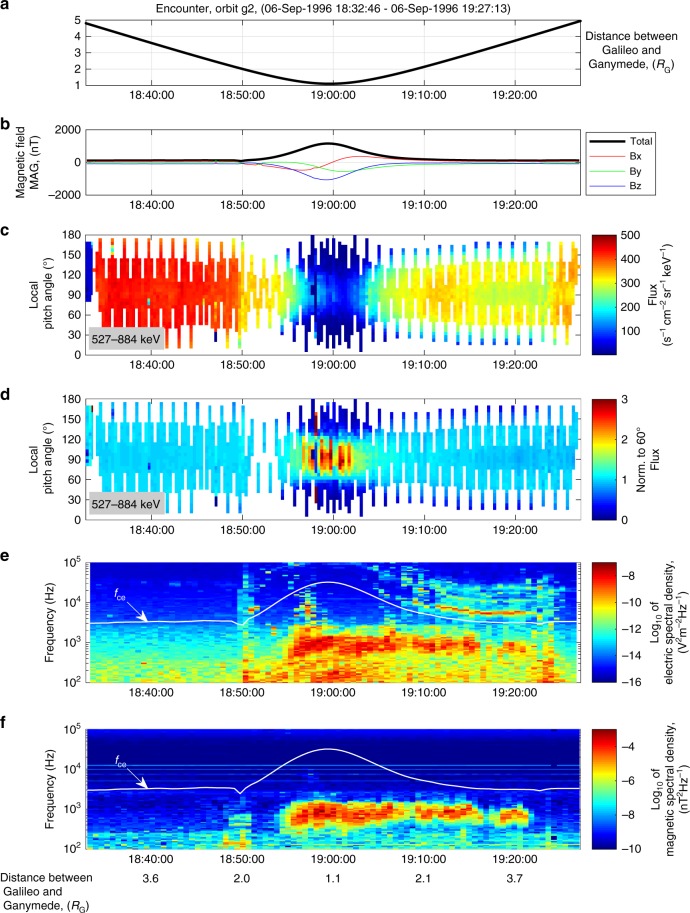
Comparison of wave power and pitch angle distributions for the encounter on 6 September 1996 (orbital segment G2). **a** The distance between Ganymede and the Galileo spacecraft, **b** measurements of the magnetic field, **c** pitch angle distribution of 527–884 keV electron fluxes, **d** same as **c** but normalized by the value of fluxes at 60° local pitch angle, **e** dynamic spectrogram of electric field very low frequency (VLF) spectral density, **f** dynamic spectrogram of magnetic field VLF spectral density

To understand the source of free energy for the waves, we have performed an analysis of the particle distributions and electric and magnetic wave measurements. We use measurements from the Galileo Energetic Particles Detector (EPD)^14^. EPD provides full angular coverage and spectral measurements of electrons from 15 keV to 1 MeV, for ions from 20 keV to 55 MeV and for heavy ions from helium through iron from 10 keV nucl^−1^ to 15 MeV nucl^−1^. Figure 2 shows particle distributions and wave measurements during the G2 encounter (see discussion of other flybys in Supplementary Note 1 and Supplementary Figures 2–4 showing G8, G28, and G29 encounters). Near the closest approach to the moon (Fig. 2a, b) the flux of electrons decreases for all pitch angles- the angle between the velocity of particles, and the magnetic field. Figure 2d presents the distribution of electrons normalized to 60° pitch angle. The normalization allows us to focus on the anisotropy of electron distributions which can generate waves as the electron distribution becomes more isotropic. While the net flux decreases at all pitch angles, Fig. 2 shows that the anisotropy for the electron distributions significantly increases, when the waves power is increased, consistent with previous observations^15^. Anisotropy may provide significant free energy which could excite the waves^16,17^. The simultaneous appearance of the waves and the anisotropy can be seen on Fig. 2d, but it is less obvious due to large temporal variability in unnormalized pitch angle distribution in Fig. 2c. During the flyby, Ganymede field lines are connected to Jupiter field lines^18,19^. Simultaneous observations of waves and charged particle distributions show that waves extend beyond the region of high anisotropy up to ~0.15 *R*_J_ (or ~4 *R*_G_, or ~10,000 km) (Fig. 2c).

## Discussion

This study shows that the presence of an obstacle embedded in the magnetic field of a larger scale that has a trapped plasma population results in the generation of waves. However, the presence of a satellite, or a satellite with a magnetic field is not sufficient to generate waves. Galileo observations of waves near Io (6 *R*_J_) and Callisto (26.3 *R*_J_), did not show strong whistler waves. In the vicinity of Io high plasma density results in a very high ratio of plasma to gyrofrequency which creates unfavorable conditions for the local acceleration of electrons by waves. In the magnetosphere of Saturn, strong waves were also observed in the vicinity of Rhea^10^ and Enceladus^20^. It was also argued that waves may be responsible for depleting electrons in the vicinity of Rhea^10^. In a similar way, waves can produce local acceleration if the plasma environment is preferential for energy diffusion. The intensity of the waves may depend on a number of parameters such as the amplitude of the magnetic field, ratio of plasma to gyro-frequency, external magnetic field and the presence of neutral particles. Note that enhanced wave activity may be also produced by objects that do not have an internal magnetic field^10,20^. However, the internal magnetic field will increase the size of the obstacle for the particles streaming near the object, resulting in the generation of stronger waves. Finding exact scaling laws and predicting maximum amplitudes that may result from non-linear saturation should be a subject of future research. Follow-up studies should verify that depleted pitch angle distributions contain sufficient free energy for the excitation of waves.

Since whistler mode waves propagate in a cone of angles about the direction of the background field, and field and density gradients refract the waves, it is likely that some waves are able to escape from field lines threading Ganymede and Jupiter. By analogy with the Earth, whistler mode waves are also reflected at the lower hybrid resonance frequency and thus waves could return to the equatorial region on a different field line after reflection at higher Jovian latitudes, provided Landau wave damping is not severe. It is therefore possible that the moons provide a source of waves that is not just confined to the magnetosphere of Ganymede but could extend outside the region. Future ray tracing studies should be focused on the propagation and reflection of waves.

If the waves escape the moons’ magnetosphere, the moons may have a pronounced effect on the radiation environment of Jupiter’s magnetosphere which may in turn affect the particle environment through wave–particle interactions. Even though the increase in wave power cannot be seen outside of ~2 moon radii, waves may be efficiently accelerating particles that are later transported radially. The increase in wave intensity that we observe may be already moderated by the wave–particle energy exchange. In other words, by the time waves are observed, they may have already given a significant portion of energy to the charged particles. These waves may allow the transport of energy from low energy and low pitch angles that are lost to Ganymede’s loss cone, and generate waves that will accelerate high energy particles.

Comparative studies of the radiation and wave environments are important for understanding the dynamics of the radiation belts, of the outer planets and Earth, and understanding of fundamental physical processes in the radiation belts such as acceleration and scattering. Comparative studies may also help us to understand the critical parameters and scaling laws that determine the behavior of plasma populations in the solar system and beyond.

This study is particularly relevant to the ongoing Juno and upcoming JUICE missions which will allow us to build a more detailed picture of these waves and their interactions with particle populations of the Jovian magnetosphere. The Juno mission reached Jupiter’s magnetosphere on July 4, 2016 and will focus on the exploration of Jupiter’s deep atmosphere and magnetosphere, while the later scheduled European Space Agency JUICE mission will have a particular emphasis on Ganymede, and a few flybys of Europa. The planned NASA Europa Clipper mission may fly by Europa >45 times. This study may additionally help with NASA’s next planned discovery mission, “Psyche”, that will explore the origin of planetary cores by studying the heaviest known M-type asteroid 16 Psyche that may potentially have a magnetic field. This study may be also relevant to laboratory studies where objects embedded in the streaming plasma may produce strong waves as well as acceleration or loss of charged particles.

Statistical observations of waves presented in this study indicated that the processes of wave generation observed for the Jovian moons embedded in the magnetosphere of Jupiter are universal and should occur for other astrophysical objects, e.g., that are stellar magnetic fields embedded in the interplanetary medium, magnetospheres of the exoplanets and magnetospheres of the moons of exoplanets. The increase in chorus wave power in the magnetospheres of exoplanets may provide free energy for the acceleration of electrons to ultra-relativistic energies. The intense synchrotron radiation from such electrons may aid in the detection of the magnetospheres of such objects.

## Methods

### Statistics of chorus waves

For the statistics of chorus waves, we follow the methodology developed in recent studies^13,21^. The data are binned in the *R*_0,_ magnetic latitude (λ), and Magnetic Local Time (MLT). The *R*_0_ of a particular point is the distance from the center of Jupiter (measured in Jovian radii) at which the magnetic field line through that point crosses the equator. The minimum frequency considered was selected according to the observations of the low frequency noise. This so-called noise floor was <200 Hz for the first 10 orbits and increased to ~350 Hz for orbits 10–15. After 15 orbits, the noise floor value was increased again and was set up between 600 and 2700 Hz due to increased noise levels of the magnetic receivers. The upper band chorus (whistler mode chorus waves above one half of the gyro-frequency) is confined to three frequency bins due to spacecraft interference at higher frequencies. The wave magnetic intensity, *P*_*B*_, is proportional to the square of magnetic amplitude *B*^2^ (nT^2^). From the measured magnetic spectral density, χ(*f*), in units of nT^2^/Hz, over a range of frequencies, we determine the power of the magnetic field for a particular frequency interval *B*^2^(*β*_i_) (measured in nT^2^), for a particular relative frequency interval, *β*_i_, by integrating over the frequency. The typical cadence for magnetic field power is Δτ~32 s. We refer to the sum of these integrations as *P*_Bi_. To calculate *P*_*B*_(*β*), we first calculate the mean value of *P*_*B*_ within any selected interval (ΔR0, ΔMLT, Δλ) by averaging all *P*_Bi_ within that spatial bin during the considered time interval. For a specific ΔR0, by calculating averages over all values of MLT, and all values of λ, we can then fit *P*_*B*_(*β*) to a power law *P*_*B*_(*β*) = *P*_o_ 10^mβ^ and determine optimal parameters of the fit *P*_o_, *m*, and *β*.

For calculations of *P*_*B*_ vs *β*, we obtain the average of all *P*_Bi_ for each relative frequency bin within a spatial bin. For calculations of *P*_*B*_ versus spatial coordinates, we obtain the average of all values of *P*_*B*_ for either the lower or upper bin within a spatial bin as required for the quasilinear calculation of the scattering rates^22,23^.

In order to perform the magnetic mapping of the locally measured values of *f*_c_ and *f*_p_ (plasma frequency) to the equator, we use the VIP4 magnetic field model with current sheet^24,25^ for Jupiter. The plasma density is measured locally by observation of the upper hybrid resonance frequency $f_{uh} = \sqrt {(f_p^2 + f_c^2)}$where *f*_c_ is the local gyrofrequency and *f*_p_ is the local plasma density. However, when the upper hybrid density is not observed, we use the analytic density model^26^. The model values of the magnetic field and plasma density at the equator are scaled by the locally measured values.

### Galileo plasma wave investigation

For Jupiter wave measurements, we use data from the Galileo Plasma Wave Investigation (PWI)^27^. This instrument provides the low-time resolution survey of magnetic and electric field data that were acquired by the low, medium, and high-frequency receivers. The electric dipole antenna is 6.6 m, and is perpendicular to the satellite spin axis. Also perpendicular to the spin axis are two magnetic search coils that are orthogonal to each other. The medium frequency receiver, MFR (42.1 Hz–160.8 kHz), was the principle receiver. The time resolution can be either 18.67 or 37.33 s with resolution Δ*f*/*f* ~ 8%. The magnetic search coils substantially degraded after the first 10 Galileo orbits. For orbits 11–34, only the electric field measurements were used in this survey and the magnetic power was estimated indirectly from the measured cold plasma refraction index *n*(*f*, *f*_c_, *f*_p_) as defined in Stix^28^. For orbits 11–34, to infer magnetic field *B*, we have used *B* = *E* *n*/*c*, where *E* is obtained from the measured electric field spectral density. Lack of magnetic field measurements is another reason why *f*_min_ was increased at this time.

To avoid spacecraft and instrumental interference, as well as natural wave interference, a number of constraints were placed on the data analyzed in this survey. Among the more important for the Galileo PWI data are the following constraints set by visually examining the observations. First, a variable minimum frequency was set to avoid instrumental noise and low frequency interference. Second, variable minimum thresholds for electric and magnetic spectral density (dependent on time and frequency) were set up to avoid background noise (due to instrument calibration, spacecraft and instrument interference, and receiver degradation). These values lie in the range 10^−13^ to 10^−11^ V^2^ m^−2^ Hz^−1^ (electric) and 10^−9^ to 10^−7^ nT^2^ Hz^−1^ (magnetic).

### Data availability

All Galileo data used in this study is publicly available. EPD data are provided at http://sd-www.jhuapl.edu/Galileo_EPD/. Wave data is available upon request from the NASA Planetary Data System archive or upon request from the authors.

## Electronic supplementary material

Supplementary Information
