# Supplementary material for: Strong whistler mode waves observed in the vicinity of Jupiter’s moons
Source: Nat Commun. 2018 Aug 7;9:3131. doi: 10.1038/s41467-018-05431-x (PMC6081473; doi:10.1038/s41467-018-05431-x)
Supplement: Supplementary file 1 — Supplementary Information [file 41467_2018_5431_MOESM1_ESM.pdf]

Supplementary materials to:

Strong whistler mode waves observed in the vicinity of Jupiter's moons

Y. Y. Shprits[1,2] *, J. D. Menietti[3] *, A. Y. Drozdov[4], R. B. Horne[5], E. E. Woodfield[5], J. B. Groene[3], M. de Soria-Santacruz[6], T. F. Averkamp[3], H. Garrett[6], C. Paranicas[7], and D. A. Gurnett[3]

1. Helmholtz Centre Potsdam, GFZ German Research Centre for Geosciences, Potsdam, Germany
2. Institute for Physics and Astronomy, University of Potsdam, Potsdam, Germany
3. Department of Physics and Astronomy, University of Iowa, Iowa City, IA, United States
4. Department of Earth, Planetary, and Space Sciences, University of California, Los Angeles, CA, United States
5. British Antarctic Survey, Cambridge, United Kingdom
6. Jet Propulsion Laboratory, California Institute of Technology, Pasadena, CA, United States
7. Applied Physics Laboratory, Johns Hopkins University, Laurel, MD, United States

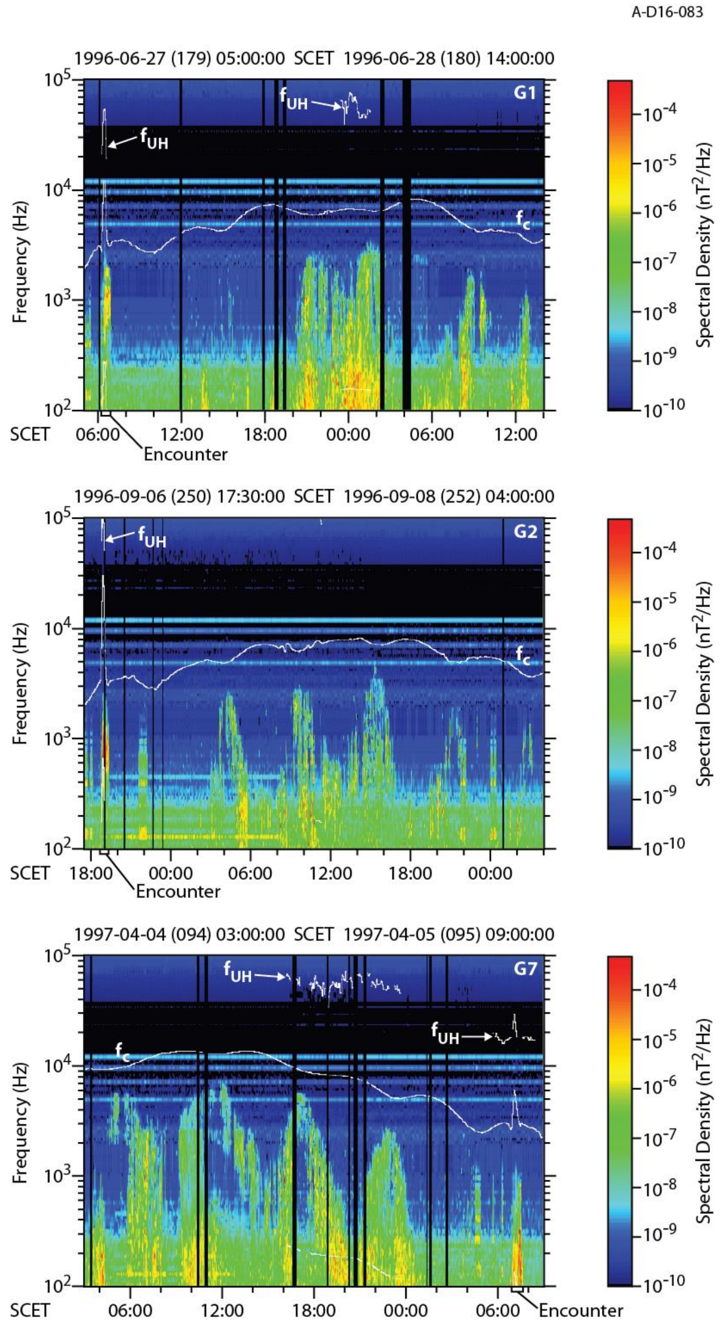

Supplementary Figure 1: Spectrograms of magnetic spectral density for three Ganymede orbital segments a) G1, b) G2, and c) G7) which contain the encounter period (shown on each panel) and many adjacent hours of often intense whistler mode emission. The encounter periods for G2 and G7 display spectral density levels at times about an order of magnitude greater than any nearby emission. The white solid line shows electron gyro-frequency and upper hybrid frequency.

Supplementary Note 1

Supplementary Figure 1 shows dynamic spectrograms during flybys G1, G2 and G7, with encounters marked on the figure. Chorus wave power, just below local gyrofrequency, dramatically increases during the encounters, exceeding typical values observed during other orbits that are also shown on Supplementary Figure 1. The wave increases are highly localized, confirming that the increase in wave power is related to the proximity to the moon. There is no visible increase in particle fluxes during flybys, which can be due to the fact that loss near the moon dominates acceleration, or that waves are in fact enhancing loss and not acceleration of particles.

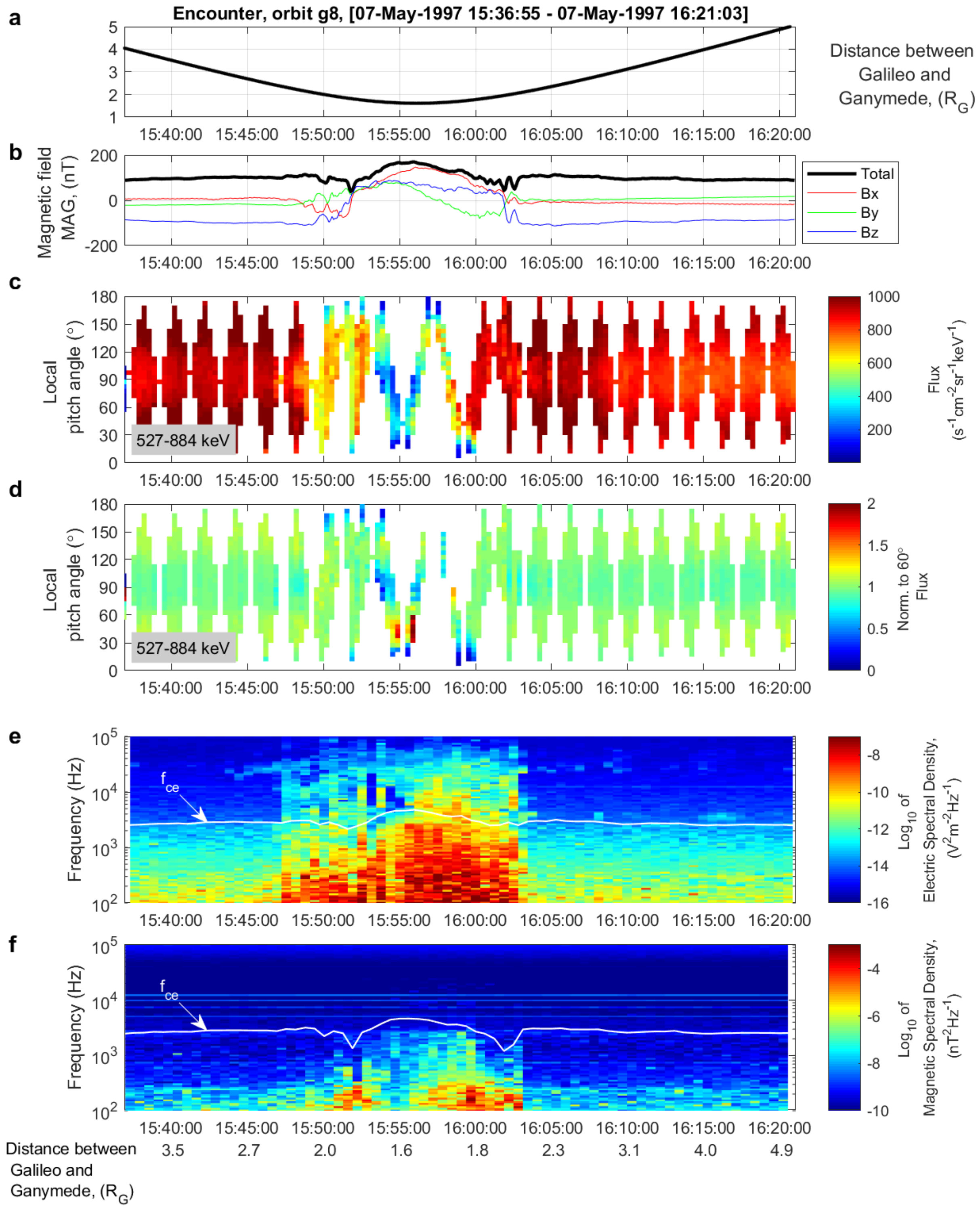

Supplementary Figure 2: Comparison of wave power and pitch angle distributions for the encounter on 7 May 1997 (orbital segment G8). Similar to Figure 2.

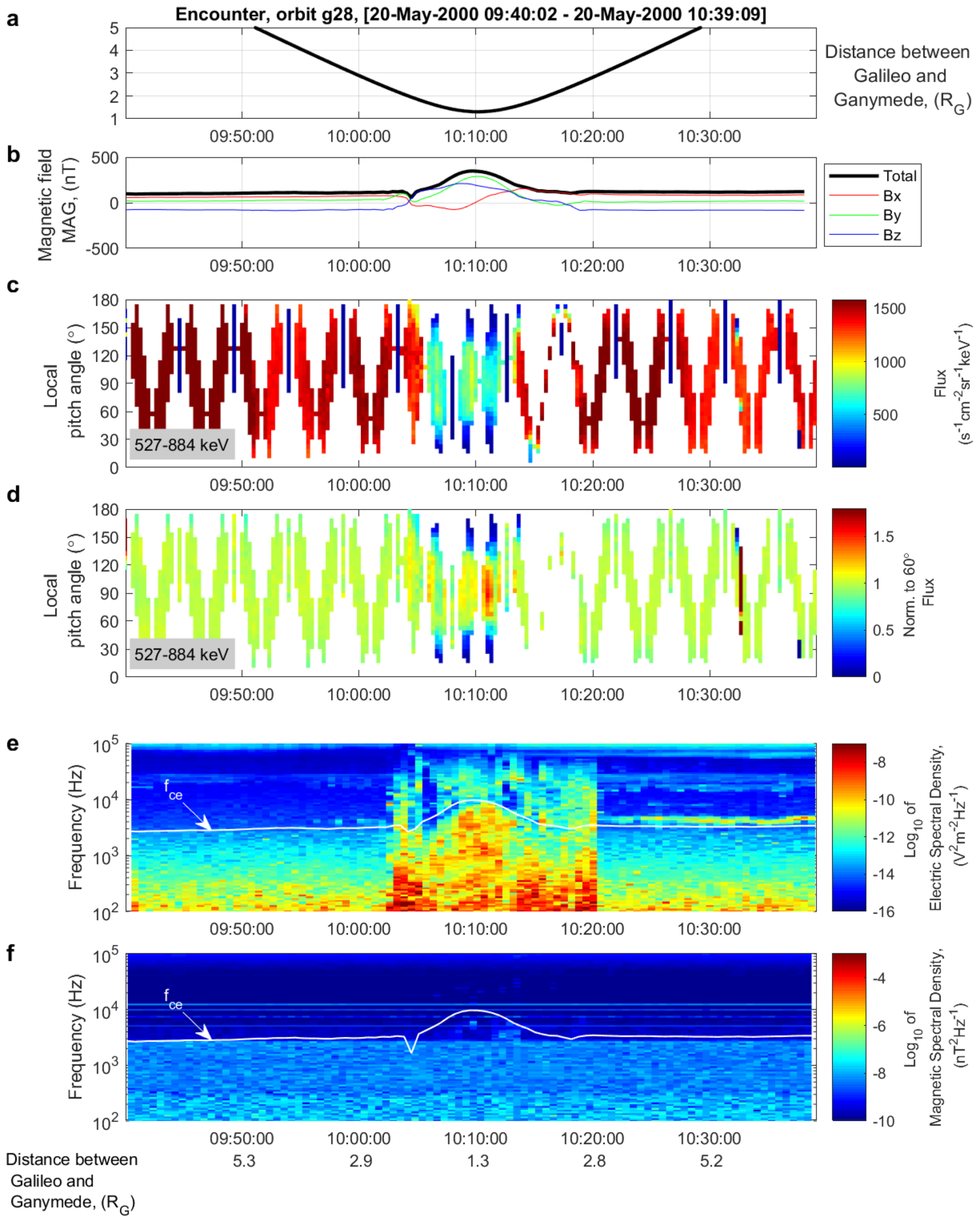

Supplementary Figure 3: Comparison of wave power and pitch angle distributions for the encounter on 20 May 2000 (orbital segment G28). Similar to Figure 2.

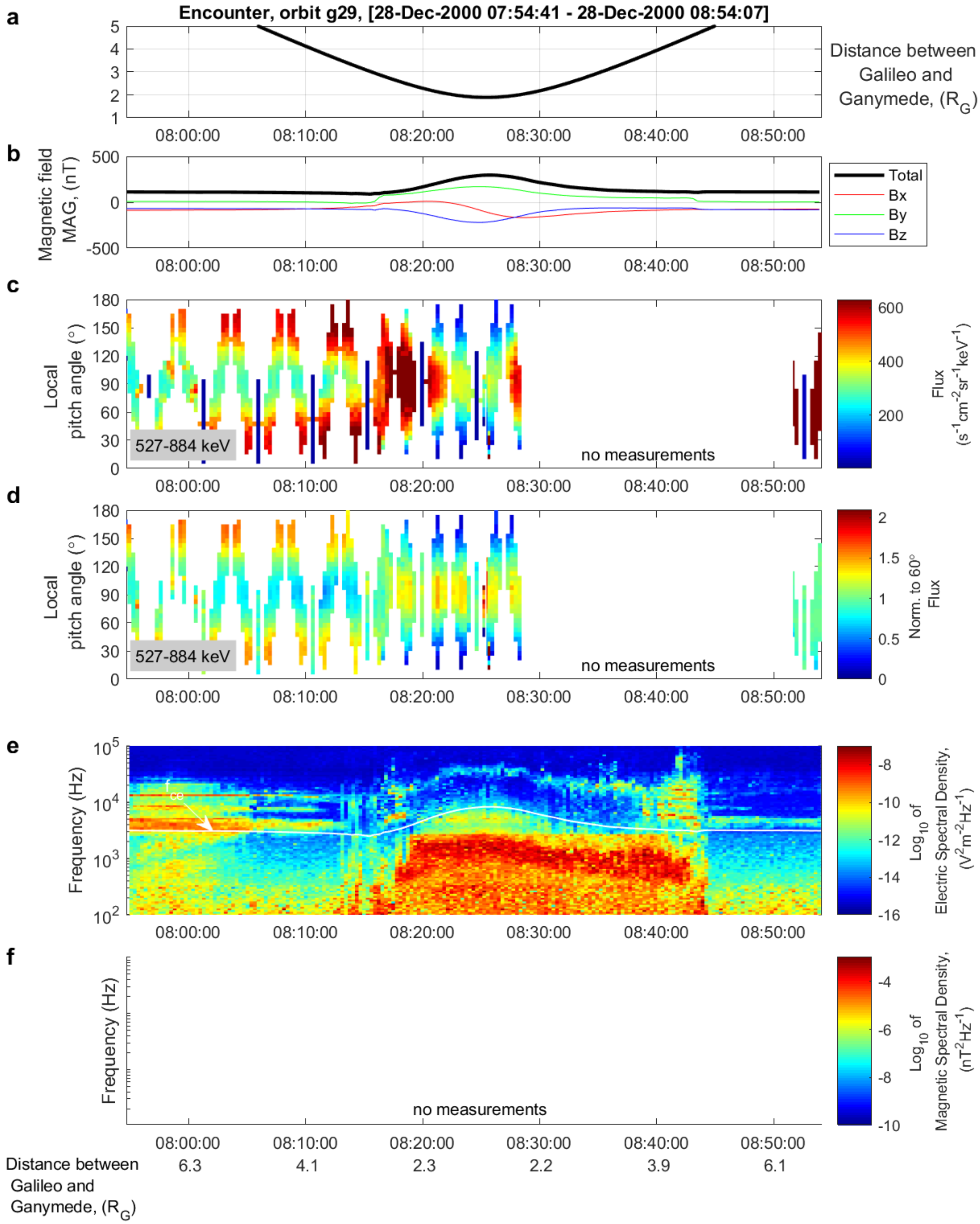

Supplementary Figure 4: Comparison of wave power and pitch angle distributions for the encounter on 28 December 2000 (orbital segment G29). Similar to Figure 2.

Supplementary Discussion

Importance of this study for exoplanet research

Remote sensing allowed the discovery of the radiation belts of Jupiter by mapping the synchrotron radiation from around 1.4 R_J. If exoplanets radiate synchrotron radiation with the same intensity as Jupiter, the radiation would not be detectable with current levels of detection technology. However, if exoplanetary systems have a strong magnetic field and internal sources of heating, synchrotron radiation may reach detectable levels. In general, acceleration of electrons inside planetary systems may be estimated from the conservation of the first adiabatic invariant and will be proportional to the magnetic field of a planet and seed population of particles provided by the stellar wind. However, VLF waves generated inside the magnetospheres may provide an additional source of heating. Such a source operates inside the Earth's magnetosphere. As argued in this manuscript, satellites inside planetary systems may work as an additional source of VLF waves that may help to accelerate electrons to ultra-relativistic energies, at which synchrotron radiation is generated by most energetic particles. Synchrotron radiation has not been yet detected for the exoplanetary systems, but may be detectable in the future. Future research may focus on obtaining realistic estimates of how high the intensity of such radiation can potentially be, and how sensitive measurement technology should be to detect it.

Parameters that determine the effect of waves on particle populations

The processes of generation of waves, propagation of waves and wave particle interactions depend on a number of different factors. A stronger magnetic field of an object would create a larger obstacle which will likely result in larger anisotropy. An abundance of particles in the near object environment will certainly be required to provide free energy for the excitation of waves.

For the waves to have a significant effect on the trapped electron population of Jupiter, waves would need to propagate into the region where particles are trapped by the magnetosphere of Jupiter. It is difficult to definitively show that such propagation of waves occurs in the magnetosphere of Ganymede, as observations are limited to a few satellite passes, and detailed observations of the wave environments around Ganymede and Europa are not available. From the observations of the terrestrial magnetosphere, we know with certainty that waves do cross field lines and that such propagation does occur. It can be facilitated by the density ducts that are often observed in the Earth's magnetosphere, which are likely to also be present in the magnetospheres of Jupiter and other objects.

Waves can accelerate electrons to higher energies (relativistic or ultra-relativistic) or can also scatter electrons in pitch angle into the loss cone and produce loss. The balance between these two processes depends on a number of factors. The most important factor in determining the net effect of acceleration and loss is whether waves are in first order resonance with near-the-edge of the loss cone particles. For example, in the Earth's magnetosphere, chorus waves on the day-side can extend up to ~40 degrees and can be in first order resonance with 1 MeV electrons, while on the night-side waves are more confined to the equator. Day-side waves produce a net loss of electrons while night side waves produce more acceleration [Shprits et al., 2009]. In the Earth's magnetosphere the net effect on 1 MeV is acceleration. However, lower energy particles (e.g. 100 keV) can be in first order resonance at small pitch angles close to the equator, which produces a very efficient loss of particles. For 100s of keV, loss processes dominate over acceleration and wave-particle interactions with whistler mode waves are a significant and efficient loss mechanism.

In the absence of detailed statistical measurements of the wave environment around Jupiter's moons, and the absence of statistical measurements of the distribution of particles fluxes around the moons, making definitive estimates of acceleration or loss is challenging. If we assume that even if 1% of waves propagate into the region of trapped population, the increase in wave power may be by a factor of 10^4 higher than without the presence of moons. Such an increase in wave power will produce 10000 times faster loss or acceleration in the vicinity of the moons, which is likely to significantly change the environment of a gas giant.
